# Sleep Duration as a Risk Factor for Cardiovascular Disease- a Review of the Recent Literature

**DOI:** 10.2174/157340310790231635

**Published:** 2010-02

**Authors:** Michiaki Nagai, Satoshi Hoshide, Kazuomi Kario

**Affiliations:** 1 Division of Cardiovascular Medicine, Department of Medicine, Jichi Medical University School of Medicine, Yakushiji, Shimotsuke, Tochigi, Japan; 2 Shobara City Soryo Clinic, Shobara, Japan, Shimoryoke, Soryo, Shobara, Hiroshima, Japan

**Keywords:** Sleep duration, hypertension, coronary heart disease, diabetes mellitus.

## Abstract

Sleep loss is a common condition in developed countries, with evidence showing that people in Western countries are sleeping on average only 6.8 hour (hr) per night, 1.5 hr less than a century ago. Although the effects of sleep deprivation on our organs have been obscure, recent epidemiological studies have revealed relationships between sleep deprivation and hypertension (HT), coronary heart disease (CHD), and diabetes mellitus (DM). This review article summarizes the literature on these relationships. Because sleep deprivation increases sympathetic nervous system activity, this increased activity serves as a common pathophysiology for HT and DM. Adequate sleep duration may be important for preventing cardiovascular diseases in modern society.

## INTRODUCTION

In the 1980s, at the peak of the Japanese economic boom, work exhaustion and sleep deprivation were blamed for large number of deaths. Most cases of “karoshi”, or “death from overwork”, involved acute cardiovascular events [1]. Likewise in the United States, a recent National Sleep Foundation poll [2], found that many Americans have long-term sleep deprivation. Only about one-third of the population (37%) reported getting 8 hour (hr) of sleep per night, and 31% reported 6 hr or less [3]. Sleep loss is a common condition in modern society, with evidence showing that people are sleeping on average only 6.8 hr per night, 1.5 hr less than a century ago [4, 5].

Although the effects of sleep deprivation on the organs have been obscure, recent studies revealed relationships between sleep deprivation and hypertension (HT), coronary heart disease (CHD), and diabetes mellitus (DM). This article reviews the literature on these relationships. 

Pickering have reviewed mainly the relationship between sleep duration and hypertension elsewhere, and observed an independent association of sleep duration to incidence of hypertension. This review will focus on the role of sleep duration, in particular short sleep duration, as a risk factor for development of hypertension, coronary heart disease, and diabetus mellitus. To set the stage, we summarized current insights in the epidemiology for these relationships, and updated the possible pathophysiology in the association of sleep duration with HT, CHD, and DM [6].

From the standpoint of sleep quality, sleep apnea syndrome should be taken into account. However, some excellent review articles have already summarized the effects of sleep apnea syndrome on HT [7], CHD [8], and DM [9].

## SLEEP DURATION AND MORTALITY

In the Alameda County Study, Wingard and Berkman [10] investigated the mortality risk associated with different sleeping patterns based on date from a 9-year mortality follow-up of 6928 adults. The analysis indicated that mortality rates from ischemic heart disease, cancer, stroke, and all causes combined were lowest for individuals sleeping 7 or 8 hr per night. Men sleeping 6 hr or less, or 9 hr or more, had 1.7 times the total age-adjusted death rate of men sleeping 7 or 8 hr per night. The comparable relative risk for women was 1.6. In prospective epidemiologic data from the American Cancer Society [11], men who reported that they usually sleep less than 4 hr were 2.80 times as likely to have died within 6 years as men who reported 7.0 to 7.9 hr of sleep. The ratio for women was 1.48. Men and women who reported sleeping 10 hr or more had about 1.8 times the mortality of those who reported 7.0 to 7.9 hr of sleep. The relationship between sleep duration and mortality was U-shaped.

## EFFECTS OF SLEEP DURATION ON HT, CHD, AND DM (TABLE 1)

### Sleep Duration and HT

The prevalence of HT has increased over the past decade despite improvements in awareness, treatment, and control of this disease [12]. During the same period, the average sleep duration in the United States has steadily declined [13].

In the First National Health and Nutrition Examination Survey (NHANES), Gangwisch *et al*. [14] conducted longitudinal analyses for 8 to 10 years on 4810 subjects from 25 to 74 years of age to determine whether or not short sleep duration increased HT incidence. Of these, 647 subjects were diagnosed with HT in that period. Sleep duration of ≤ 5 hr per night was associated with a significantly increased risk of HT in subjects between the ages of 32 and 59 years. However, this significant relationship was not found in subjects between aged of 60 to 86 years.

This result would suggest a strong association between short sleep duration and the development of HT, especially in middle-aged subjects.

#### Mechanism Underlying the Relationship between Sleep Duration and HT 

**Fig. (1):**
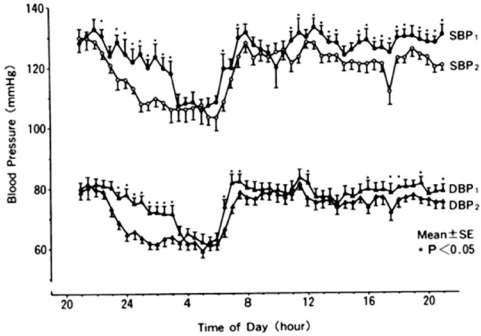
Means of ambulatory blood pressure on a normal workday and a sleep-insufficient day. BP indicates blood pressure; SBP1, systolic BP on sleep-insufficient day; SBP2, systolic BP on normal workday; DBP1, diastolic BP on sleep-insufficient day; DBP2, diastolic BP on normal workday. (From Tochikubo *et al*. [16]. Hypertension 1996; 27: 1318-1324).

Short sleep duration was associated with the development of HT. This may be attributable in part to autonomic dysregulation that changes the predominant neutral interaction of sympathovagal balance during sleep into increased sympathetic tone. In 24 hr ambulatory blood pressure monitoring (ABPM) studies, BP tended to rise the day after sleep deprivation in both normotensives [15] and hypertensives [16]. Similarly, Tochikubo [17] *et al*. showed significant increases in BP, low-frequency/high-frequency (LF/HF) ratio, and urinary excretion of norepinephrine, especially in the evening, the day after sleep deprivation in 18 male technical workers aged 23 to 48 years (Fig. **1**). These data suggest that a lack of sleep may increase sympathetic nervous system activity the next day. Zhong *et al*. [18] assessed cardiovascular autonomic modulation during 36 hr of total sleep deprivation in 18 normal healthy subjects. LF was significantly increased at 12 and 24 hr, as was LF/HF at 12 hr, whereas HF was decreased at 12 hr of sleep deprivation.

Using polysomnography, Irwin *et al*. [19] examined whether or not nocturnal vagal tone indexed by HF was related to measures of sleep depth and daytime perceptions of sleep quality, sleepiness, and fatigue in alcohol-dependent patients. Compared with the controls, these patients showed decreses in delta sleep time along with impairments in sleep quality, daytime energy, and the HF component both before and during sleep.

Irwin and Ziegler [20] investigated whether or not sleep deprivation induces differential cardiovascular and sympathetic responses, and measured heart rate, BP, and circulating sympathetic catecholamines in 36 abstinent alcohol-dependent men and 36 age-, gender-, and ethnicity-matched controls. Although baseline heart rate, BP, and sympathetic catecholamines were similar in both groups, partial night sleep deprivation induced greater increases in heart rate and circulating levels of norepinephrine and epinephrine in the alcohol-dependent men than in controls.

Vascular lesions with atherosclerosis are filled with immune cells that effect inflammatory responses. These responses are suggested to initiate plaque activation that progress hypertensive status. Born *et al*. have shown that stimulated ex vivo production of interleukin-2 (IL-2) is higher during sleep suggesting that this effect is dependent on sleep [21, 22]. Conversely, partial night sleep deprivation induced decreases in stimulated production of IL-2 and natural killer cell responses [23, 24]. 

Irwin *et al.* [25] measured circulating levels of catecholamines and IL-2 sampled every 30 minutes during two nights: undisturbed, baseline sleep and partial sleep deprivation-late night (PSD-L; awake from 0300-0600) in 17 healthy male volunteers. On the PSD-L night, levels of norepinephrine and epinephrine significantly increased in association with nocturnal awakening. Nocturnal levels of circulating IL-2 did not change with sleep onset or in relation to PSD-L or various sleep stages.

Elevated plasma concentrations of C-reactive protein (CRP) are indicative of systemic inflammation. Circulating CRP levels are representative marker of vascular damage progression. In this point, higher CRP level would be associated with incidence of HT. Meier-Ewert *et al*. [26] measured high-sensitivity C-reactive protein (hs-CRP) collected every 90 minutes for 5 consecutive days in 10 healthy adults who stayed awake for 88 continuous hours. The hs-CRP concentrations and systolic BP increased during that period.

Additionally, excessive waking periods would induce long-standing psycho-social stress. Recently, stress exposure has been found to lead to increased salt intake and inhibition of renal salt excretion [27]. These processes may be related to HT development during volume overload for 24 hr and to arterial remodeling.

In short, sleep deprivation is associated with increased sympathetic tone. Sleep loss might serve to elevate nocturnal catecholamine levels and contribute to cardiovascular disease.

#### Sleep Duration and CHD

Short sleep duration imposed on a group of healthy subjects increased sympathetic nervous system activity and blood pressure elevation. Therefore sustained short sleep duration could lead to adverse cardiovascular consequence.

A prospective study in the United States showed that the standardized mortality ratio of CHD was highest among those who worked 67 hr or more a week [28]. Case-control studies in The Netherland [29], Denmark [30], and Sweden [31] also reported that prolonged working time was associated with an increased risk of acute myocardial infarction (AMI). Another case-control study in Japan found significantly increased odds ratios of AMI for those who worked more than 11 hr a day [32].

It is possible to think that sleep deprivation caused by overtime work is associated with an increased risk of AMI. The American Cancer Society Study showed that men sleeping 4 hr or less had higher mortality from CHD than those sleeping 7-7.9 hr [11]. The Alameda County Study [10] and the study by Partinen *et al*. [33] also noted that men who slept less than 6 hr had a greater risk of developing CHD than men sleeping 7-8 hr.

In a Japanese case-control study, Liu *et al*. [34] examined the relationships between the risk of AMI and both work hours and sleep duration. The cases were 260 men aged 40-79 admitted to hospitals with AMI during 1996-8. The controls were 445 AMI-free men matched for age and residence who were recruited from the resident registers. Longer working hours were related to shorter hours of sleep, and more days a week with less than 5 hr in both AMI patients and normal healthy subjects. Working more than 60 hours per week was related to increased incidence of AMI; those working such hours were twice as likely to develop AMI than those working no more than 40 hours. Those who slept no more than 5 hr per night had a 2.3-fold greater risk of AMI than those getting 6-8 hr sleep.

In the Nurses’ Health Study, Ayas *et al*. [35] investigated the relationship between self-reported sleep duration and the incidence of CHD in 71,617 female health professionals aged 45-65 years in the United States. A total of 934 coronary events were documented (271 fatal and 663 nonfatal) during the 10 years of follow up. The age-adjusted relative risks of CHD, with 8 hr of daily sleep being considered the reference group, for individuals reporting 5 or fewer, 6, and 7 hr of sleep were 1.82, 1.30, and 1.06, respectively. The relative risk for 9 or more hr of sleep was 1.57.

These reports support the notion that short or long sleep duration is independently associated with an increased likelihood of coronary events. There is thus a U-shaped relationship between sleep duration and CHD incidence.

Several biological explanations are possible for the increased risk of CHD associated with sleep deprivation. Hypertension and increased sympathetic nervous system activity may underlie this relationship. Sleep deprivation also increases sympathetic nervous system activity, heart rate, and vasoconstriction as well as salt retention. These factors may be associated with hypertension caused by cardiac overdrive and volume overload.

On the other hand, there have been few reports on the relationship between long sleep duration and CHD. Sleep apnea syndrome would be a candidate factor for this relationship, but there is little evidence as to whether patients with sleep apnea have long sleep duration or not.

#### Sleep Duration and DM

Spiegel *et al*. [36] assessed the activity of the hypothalamo-pituitary-adrenal axis and sympathovagal balance in 11 young men under two different sets of conditions: after their time in bed had been restricted to 4 hr per night for 6 consecutive nights, and after a sleep-recovery period when the participants were allowed 12 hr in bed per night for 6 consecutive nights. Lower glucose tolerance, higher evening cortisol concentration, and increased activity of the sympathetic nervous system occurred in the sleep deprivation experiment than in the fully rested condition. Similar results, that sleep deprivation was associated with lower glucose tolerance and increased insulin resistance, were found elsewhere [37].

Whereas these results were based mainly on an experimental sleep restriction, the metabolic effects of habitual sleep restriction were reported recently. In the Sleep Heart Health Study (SHHS) [38], 722 men and 764 women, aged 53 to 93 years, were assessed to determine the cross-sectional relationship between usual sleep time and both DM and impaired glucose tolerance (IGT). Compared with those sleeping 7 to 8 hr per night, subjects sleeping 5 hr or less and 6 hr per night had adjusted odds ratios of 2.51 and 1.66 for DM and of 1.33 and 1.58 for IGT, respectively. Subjects sleeping 9 hr or more per night also had increased odds ratios for DM and IGT. These associations persisted when subjects with insomnia were excluded.

In the Massachusetts Male Aging Study (MMAS) [39], a cohort of men without DM at baseline were followed for the development of DM for 15 years. When those reporting 7 hr of sleep per night served as the reference group, men reporting less sleep (≤5 and 6 hours sleep per night) were twice as likely to develop DM, and men reporting long sleep duration (>8 hours of sleep per night) were more than three times as likely to develop DM during the follow-up period. The risk elevation remained essentially unchanged after adjustments for age, hypertension, smoking status, self-rated health status, education, and waist circumference.

Short or long sleep duration increases the risks of developing DM, independent of confounding factors. This suggests that U-shaped associations exist between sleep duration and the incidence of DM.

Short sleep duration was associated with disruption of endogenous system via increased sympathetic nervous system activity. Several studies have identified some agents that links short sleep duration and incidence of DM.

Experimental sleep deprivation causes elevated evening levels of cortisol that may predispose individuals to insulin resistance [36, 40]. On the other hand, recent studies have linked sleep disruption with reduced testosterone levels [41]. Low levels of testosterone have been associated with obesity [42], elevated levels of insulin and glucose [43, 44], and DM incidence [45, 46]. In the MMAS [39], not cortisol but testosterone level had considerable impact on the relationship between sleep duration and DM incidence among male subjects. In addition to this, sleep deprivation reduced levels of leptin and increased levels of ghrelin. Increased appetite was correlated with an increased ratio of ghrelin to leptin [47].

Sleep restriction results in an increase in sympathetic tone, which inhibits pancreatic function [36, 48]. Autonomic nervous system dysregulation may be also associated with the impact of sleep deprivation on the increased incidence of DM.

On the other hand, few studies have investigated the effect of long-term sleep duration on triggering DM. Sleep-disordered breathing, a known cause of daytime sleepiness, would be linked to increased sympathetic tone and glucose intolerance [7]. These mechanisms may be associated with the relationship between long sleep duration and DM.

In summary, sleep loss was associated with DM via endocrine system disruption, which would, in part, affect our eating behavior and autonomic balance

## SLEEP DEPRIVATION AND DECREASED MELATONIN SECRETION - A POSSIBLE LINK WITH THE INCREASED SYMPATHETIC NERVOUS SYSTEM ACTIVITY DURING SLEEP (FIG. 2).

Recent studies have shown that dysregulation of melatonin secretion may be associated with HT and IGT. The suprachiasmatic nucleus (SCN) in the hypothalamus, which serves as an essential component of the circadian clock, affects melatonin secretion. Light activates SCN neurons via retino-hypothalamic glutamate secretion. The SCN reaches autonomic neurons of the paraventricular nucleus (PVN) which projects to the intermediolateral column of the spinal cord (IML) where preganglionic sympathetic neurons are located that control the outflow to the pineal gland [49-51]. The activation of SCN neurons has been suggested to cause a release of a specific γ-aminobutyric acid (GABA) from their terminals in the PVN and consequently may inhibit melatonin secretion [52-54].

Injection of transneuronal tracers into various organs ranging from heart to ovaries and from white to brown adipose tissue resulted in the labeling of neurons in the SCN via the sympathetic and parasympathetic branches of the autonomic nervous system [55-58]. These data demonstrate that the SCN may transmit its circadian message, influencing the activity – rest cycle of peripheral organs, not only by the secretion of hormones that may freely pass all kinds of tissue barriers, such as melatonin, corticosterone, gonadal hormones and thyroid hormones but also by direct nervous system control of these organs. And, SCN neurons are labeled via both the sympathetic and parasympathetic systems, indicating that the SCN may indeed support both the activity and rest periods of the circadian cycle.

The SCN-PVN-autonomic axis affects hormone secretion and the sensitivity of the target organs to these hormones through neuronal mechanisms. For example, anatomical and physiological evidence shows that the SCN influences the level of insulin secretion from the pancreas [59-61]. Additionally, when the SCN stimulates glucose secretion from the liver, at the same time it stimulates glucose uptake by other tissues [61].

This timely orchestrated action of the SCN on glucose metabolism prepares the body, just before waking, for the coming period of activity. Disruption of this orchestration may be associated with the pathophysiology of HT and DM.

For a long time, physiological studies have indicated that patients suffering from these diseases exhibit disturbed circadian rhythm [62, 63]. Severe decreases in staining for several SCN neurotransmitters in hypertensive patients revealed an anatomical basis for these disturbances [64]. Interestingly, the finding that followed this observation was that, in the same patients, the activity of PVN corticotropin-releasing hormone (CRH) neurons in the PVN was enhanced, which would suggest that the SCN would have an inhibitory effect on the PVN CRH neurons. This intriguing observation suggests that SCN activity is changed by a different autonomic feedback in HT. Since the SCN anatomy was changed in spontaneous hypertensive rats (SHR), and since transplantation of the hypothalamus containing the SCN from SHR to normotensive rats induced HT, it follows that a changed SCN might precede the development of HT [64]. Furthermore, recent evidence supports the notion that circadian disturbances can be detected before the development of HT or DM [65, 66].

## HOW LONG SHOULD WE SLEEP? WHAT SLEEP MEDICATION SHOULD WE CHOOSE?

In brief, recent studies suggest that sleep deprivation is a significant risk factor for developing HT, CHD, or DM. In the evening of the day after sleep deprivation, sympathetic nervous system activity and BP are elevated. These increases may be associated with the increased risk of CHD. On the other hand, because sleep deprivation is related to reduced salt excretion or IGT, excessive intake of calories or salt should be avoided by people who are not getting enough sleep. In addition, since alcohol intake with sleep deprivation tends to elevate BP the next evening, people also should avoid drinking alcohol after not getting enough sleep.

Short sleep time is associated with increased sympathetic nervous system activity, which may point to this hypothesis that α or β adrenergic sympathetic blockade may be suitable for hypertensives who have sleep deprivation or IGT, because these sympathetic blockades have been shown to preserve pancreas function [67]. However, there is little evidence to support the notion that a sympathetic system blockade could be truly beneficial to such populations.

**Fig. (3):**
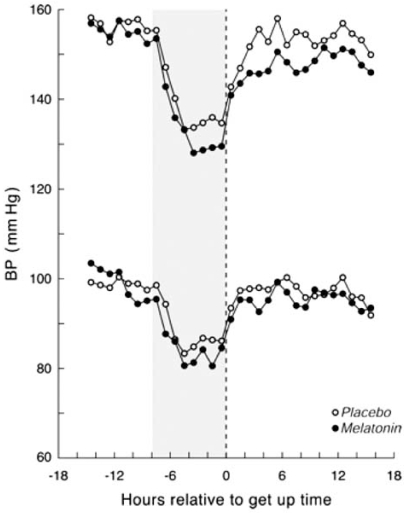
Means of ambulatory blood pressure after repeated melatonin and repeated placebo sadministration. The light-gray background indicates the average period in bed. (From Scheer *et al*. (66). Hypertension 2004; 43: 192-197).

Recently, Scheer *et al*. [68] conducted a randomized, double-blind, placebo-controlled crossover trial in 16 men with untreated essential HT to investigate the influence of acute (single) and repeated (daily for 3 weeks) oral melatonin (2.5 mg) intake 1 hr before sleep on 24 hr ABP and sleep quality. Repeated melatonin intake reduced nocturnal systolic and diastolic BP by 6 and 4 mmHg, respectively (Fig. **3**). The treatment did not affect heart rate. Repeated but not acute melatonin intake also improved sleep. These findings suggested that support of the circadian pacemaker function may provide a new strategy for the treatment of essential hypertension.

Similarly, Cagnacci *et al*. [69] investigated whether or not prolonged nocturnal administration of melatonin could influence daily BP rhythm in women. In a randomized, double-blind, placebo-controlled crossover study, 9 females with normal BP and 9 females who were treated essential hypertensives received a 3-week course of melatonin (3 mg) or placebo intake 1 hr before going to bed. ABP was measured to evaluate BP reduction with melatonin intake. In comparison with placebo, melatonin administration did not influence diurnal BP but did significantly decrease nocturnal systolic and diastolic BP, by 3.7 and 3.6 mmHg, respectively.

Additionally, Grossman *et al*. [70] studied the potency of melatonin to reduce nighttime BP. The 38 treated hypertensive patients (16 females) with nocturnal hypertension were randomized in a double-blind fashion to receive either controlled-release melatonin (2 mg) or placebo 2 hrs before bedtime for 4 weeks. Melatonin treatment reduced nocturnal systolic and diastolic BP significantly, whereas placebo had no effect on nocturnal BP (Table **2**).

Simko and Paulis [71] summarized a potential role of melatonin in antihypertensive treatment. The nighttime production of melatonin is found to be reduced in hypertensive individuals. Melatonin administration decreased BP in several animal models of hypertension, in healthy men and women, and in patients with arterial HT. The most promising results were achieved in patients with nondipping nocturnal BP. Simko and Paulis [71] considered several potential mechanisms of BP reduction in melatonin, as follows. Melatonin can, via its scavenging and antioxidant nature, improve endothelial function via the increased availability of nitric oxide, thereby exerting vasodilatory and hypotensive effects, it can also interfere with the peripheral and central autonomic nervous systems, with a subsequent decrease in the tone of the adrenergic system and an increase in the cholinergic system. 

## CONCLUSION

The recent literatures confirms that sleep deprivation is associated with HT, CHD, and DM. Increased sympathetic nervous system activity is considered to serve as a common pathophysiology in sleep deprivation’s relationships with these diseases. Especially, the relationship between sleep time and incidence of CHD or DM is U-shaped. Sleep periods that are neither too short nor too long may be important to keep us healthy. 

## Figures and Tables

**Fig. (2):**
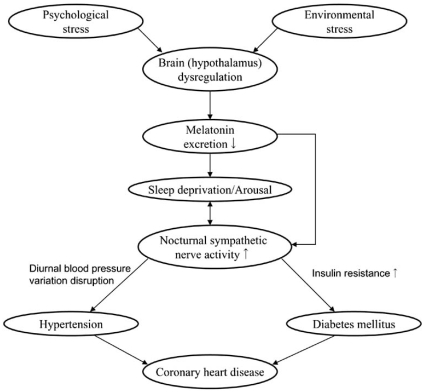
Chart for the relationships between sleep deprivation and hypertension, diabetes mellitus, and coronary heart disease.

**Table 1:** The Relationship between Sleep Duration and the Risk of Hypertension, Coronary Heart Disease, and Diabetes Mellitus

	Study or Author	Subject	Age (Year)	Follow-up period	Result
Hypertension (HT)	NHANES (2006)	4810 subjects without HT	25~74	8 - 10 (years)	Increased risk of HT in subjects with 5 or fewer hours of sleep.
Coronary heart disease (CHD)	Nurses’ Health Study (2003)	71,617 females without CHD	45~65	10 (years)	Increased risk of CHD in subjects with 5 or fewer, 6, 7, and 9 or more compared with those with 8 hours of sleep. U-shaped phenomenon (+).
	Liu *et al.* (2006)	260 males with AMI and 422 males without AMI	40~79	(-) (case- control study)	Increased odds ratios of AMI in subjects with 5 or fewer compared with those with 6 to 8 hours of sleep.
Diabetes mellitus (DM) /Impaired glucose tolerance (IGT)	SHHS (2005)	722 males and 764 females	53~93	(-) (cross-sectional design)	Increased odds ratios of DM and IGT in subjects with 5 or fewer, 6, and 9 or more compared with those getting 7 to 8 hours of sleep. ·U-shaped phenomenon (+).
	MMAS (2006)	1709 males without DM	40~70	15 (years)	Increased risk of DM in subjects with 5 or fewer, 6, and 8 or more compared with those getting 7 hours of sleep. U-shaped phenomenon (+).

**Table 2:** The Relationship between Melatonin Administration and BP Control

Author (Year)	Subjects (Age)	Melatonin	Study design	Treat period	Results
Scheer *et al*. (2004)	18 men with untreated HT (55±8 mean age)	Oral melatonin (2.5mg) intake 1hr before sleep	Randomized, Double blind, placebo controlled, crossover	3 week	Melatonin intake reduced systolic and diastolic blood pressure during sleep by 6 and 4mmHg. Day-night amplitudes of the rhythms in systolic and diastolic blood pressures were increased by 15% and 25%.
Cagnacci *et al*. (2005)	9 normotensive women and 9 women with treated HT (47 to 63 years of age)	Oral melatonin (3.0mg) intake 1hr before sleep	Randomized, double blind, placebo controlled, crossover	3 week	Melatonin intake reduced systolic and diastolic blood pressure during sleep by 3.8 and 3.6mmHg. Melatonin intake was related to the increase in the day-night BP difference.
Grossman *et al.* (2006)	38 treated HTs with nocturnal HT (22 males, 64±11 mean age)	Oral melatonin (2.0mg) intake 2hr before sleep	Randomized, double blind, placebo controlled	4 week	Melatonin intake reduced nocturnal systolic BP from 136±9 to 130±10mmHg, and diastolic BP from 72±11 to 69±9mmHg. The reduction in nocturnal systolic BP was significantly greater with melatonin than with placebo.
